# Monitoring in Real Time the Formation and Removal of Biofilms from Clinical Related Pathogens Using an Impedance-Based Technology

**DOI:** 10.1371/journal.pone.0163966

**Published:** 2016-10-03

**Authors:** Diana Gutiérrez, Claudio Hidalgo-Cantabrana, Ana Rodríguez, Pilar García, Patricia Ruas-Madiedo

**Affiliations:** Instituto de Productos Lácteos de Asturias–Consejo Superior de Investigaciones Científicas (IPLA-CSIC), Villaviciosa, Asturias, Spain; Institut Pasteur, FRANCE

## Abstract

Bacteria found in diverse ecosystems grow in a community of aggregated cells that favors their survival and colonization. Different extracellular polymeric substances are used to entrap this multispecies community forming a biofilm, which can be associated to biotic and abiotic surfaces. This widespread and successful way of bacterial life, however, can lead to negative effects for human activity since many pathogen and spoiling bacteria form biofilms which are not easy to eradicate. Therefore, the search for novel anti-biofilm bio-active molecules is a very active research area for which simple, reliable, and fast screening methods are demanded. In this work we have successfully validated an impedance-based method, initially developed for the study of adherent eukaryotic cells, to monitor the formation of single-species biofilms of three model bacteria in real time. The xCelligence real time cell analyzer (RTCA) equipment uses specific microtiter E-plates coated with gold-microelectrodes that detect the attachment of adherent cells, thus modifying the impedance signal. In the current study, this technology allowed the distinction between biofilm-producers and non-producers of *Staphylococcus aureus* and *Staphylococcus epidermidis*, as well as the formation of *Streptococcus mutans* biofilms only when sucrose was present in the culture medium. Besides, different impedance values permitted discrimination among the biofilm-producing strains tested regardless of the nature of the polymeric biofilm matrix. Finally, we have continuously monitored the inhibition of staphylococcal biofilm formation by the bacteriophage phi-IPLA7 and the bacteriophage-encoded endolysin LysH5, as well as the removal of a preformed biofilm by this last antimicrobial treatment. Results observed with the impedance-based method showed high correlation with those obtained with standard approaches, such as crystal violet staining and bacteria enumeration, as well as with those obtained upon other abiotic surfaces (polystyrene and stainless steel). Therefore, this RTCA technology opens new opportunities in the biofilm research arena and its application could be further explored for other bacterial genera as well as for different bio-active molecules.

## Introduction

Bacterial biofilms are complex communities composed of one or multiple species adhered to a solid surface and surrounded by a polymeric extracellular matrix secreted by the cells. The matrix can be composed of polysaccharides, proteins, teichoic acids, nucleic acids and lipids, which confer protection to the bacteria, physical structure and exchange of substances [1]. Inside the biofilm, gradients of nutrients and oxygen lead to differences in the physiological state of bacteria depending on their location within the biofilm. Furthermore, both biofilm development and dispersion is a fine regulated process where cell-cell communication is mediated by quorum sensing systems [2] and di-GMP levels [3]. Currently, the first step of biofilm formation involves the attachment of bacteria to biotic or abiotic surfaces in which different structural microbial molecules could be involved [4]. Biofilms are the most common life style of microorganisms in nature and bacterial biofilms are especially relevant in clinical and several industrial settings [5]. Indeed, many chronic infections are caused by pathogenic bacteria growing in biofilms [6]. This is mainly due to their inherent resistance to host defense mechanisms and to antimicrobial agents, including antibiotics and disinfectants. Biofilm structure provides a reduced diffusion of these compounds, which turn them to be ineffective. In fact, treatment of these infections is a serious challenge due to the reduced penetration of antibiotics inside the biofilm [7]. In addition, the high proportion of cells having a low growth rate and/or dormancy state also makes difficult the activity of antimicrobials [8].

Among bacteria involved in biofilm-associated infections, the species *Staphylococcus aureus* and *Staphylococcus epidermidis* are especially relevant as cause of nosocomial infections mainly linked to the colonization of implanted medical devices [9]. For these bacteria, the biofilm formation is one of the major virulence factors associated with their ability to colonize human tissues and abiotic surfaces [10]. A clear relationship has been established between production of the extracellular poly-β-(1–6)-N-acetyl-glucosamine (PIA/PNAG) polysaccharide and virulence in animal models of infection [11,12]. Biofilms of staphylococcal strains that lack PNAG in their matrix, are based on the presence of surface proteins such as Bap (biofilm-associated protein) [13] and SasG [14] or the fibronectin-binding proteins (FNBPs). The extracellular DNA (eDNA) derived from lysed bacteria is also a major component of staphylococcal biofilms [15].

Dental plaque is another example of multiple species biofilm that could have potential clinical implications due to caries occurrence following an oral-microbiota dysbiosis [16]. Streptococci seem to be involved in the initial steps of cariogenic biofilm formation [17,18]. Indeed, it has been indicated that *Streptococus mutans* is the key player in the formation of the exopolysaccharide material (glucans) that constitutes the binding matrix to stick other bacteria. Some specific glucosyltransferases (GTFs) involved in the synthesis of glucans are able to initially bind to dental pellicle, or to adsorb to other non-adherent microorganisms; using sucrose from diet as substrate, these enzymes initiate the formation of the polymeric material [19,20].

Thus, the obvious negative consequences of biofilm-forming pathogenic bacteria make the search for novel anti-biofilm molecules a very active research area. To achieve this goal, the use of fast, reliable and accurate technologies allowing the screening of active molecules is of pivotal relevance. In this work, we have assessed the suitability of an impedance-based instrument as an alternative to standard methods (crystal violet staining and bacterial viable counting), for monitoring the biofilm formation of representative biofilm-producing strains in real-time. The suitability of this method to address the effect of two anti-biofilm compounds (endolysin LysH5 and bacteriophage phi-IPLA7) on the development and removal of staphylococcal biofilms was further evaluated.

## Material and Methods

### Bacterial strains and culture conditions

Several strains of *S*. *mutans*, *S*. *aureus* and *S*. *epidermidis*, from different origin and properties, were selected as model bacteria able to form biofilms (Table 1). As standard culture conditions *S*. *mutans* was grown in BHI (Brain Heart Infusion, Oxoid, Basingstoke, Hampshire, UK) at 37°C under 5% CO_2_ atmosphere, and *Staphylococcus* spp. in TSB (Tryptic Soy Broth, Scharlau, Barcelona, Spain) with 0.25% glucose (TSBG) at 37°C with shaking. Bacteria from stocks (at -80°C) were plated onto the corresponding agar media (containing 2% agar) and incubated for 24 or 48 hours. Afterwards, single colonies were picked up to inoculate BHI or TSBG and cultured overnight; these cultures were used to inoculate (2% vol/vol) fresh media which were incubated for 18±1 h, under standard conditions, to obtain the cells for further experiments.

**Table 1:** Strains of *Streptococcus mutans* and *Staphylococcus* ssp. used in this study.

Species	Strain	Origin	Properties *	Reference
*S*. *mutans*	CI2366	Clinical isolate		[21]
	NCTC10449	Culture collection	Type strain	[21]
*S*. *aureus*	15981	Clinical isolate	PNAG producer	[22]
	ISP479r	Clinical isolate	PNAG producer	[22]
	132	Clinical isolate	PNAG producer	[23]
	V329	Bovine subclinical mastitis	Bap protein producer	[13]
	CH1368	-	Non biofilm producer	-
*S*. *epidermidis*	F12	Breast milk of lactating woman	PNAG producer	[24]
	CH48	-	Non biofilm producer	-

* PNAG (PIA/PNAG): poly-β-(1–6)-N-acetyl-glucosamine polysaccharide.

### Monitoring biofilm formation in real time

The real time cell analyzer (RTCA) xCELLigence (ACEA Bioscience Inc., San Diego, CA) equipment, based on impedance measurement, was used to monitor the formation of bacterial biofilms. This equipment has initially been developed to detect variations in the impedance signal (expressed as cell index, CI) due to the attachment and growth of adherent eukaryotic cells upon the gold-microelectrodes placed in the bottom of E-plates (ACEA Bioscience Inc.), which have a surface equivalent to 96-wells standard microplates. The RTCA-DP platform used in our work has three holder units to place three independent 16-well E-plates [25]. The equipment was introduced into incubators at 37°C (with or without 5% CO_2_) and after bacterial seeding, the CI was recorded every 10 min during the incubation period.

**Fig 1:**
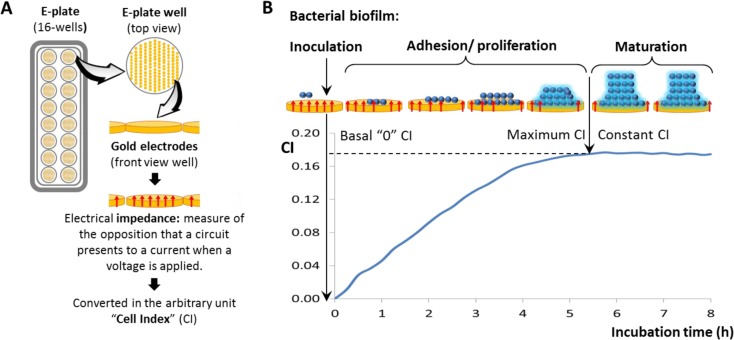
Schematic drawing of the basis to monitor bacterial biofilm formation by the xCellingence equipment. Standard E-plates (16-wells) and magnification of one of the wells coated with gold microelectrodes (**A**). Evolution of the “cell index” (CI), which derived from the electric impedance, throughout the different steps of bacterial biofilm formation by the strain *S*. *aureus* 15981 (**B**).

The underlying principle behind this method for monitoring bacterial biofilm formation is depicted in Fig 1. When the bacterial culture is added, the basal CI value is “0” because there is not opposition to the pass of the current through the gold microelectrodes placed in the well. Once that the bacteria adhere to the microelectrodes and they begin to proliferate, there is a modification of the electric impedance and the CI starts to increase. Polymeric material can be formed during this proliferative state contributing, as well, to the modification of the impedance signal. The maximum CI is reached when the opposition to the pass of the current is kept constant, indicating that the surface of the microelectrode is covered by the biofilm which has reached the stationary phase. This CI value remains constant throughout the maturation of the biofilm decreasing afterwards, once the biofilm initiates the detachment phase (step not shown).

### *S*. *mutans* and *Staphylococcus* spp. biofilm formation

Standardized *S*. *mutans* cultures (counts about 10^9^ CFU/ml) were washed twice with PBS and diluted (1/10, vol/vol) in BHI. These bacterial suspensions were used to inoculate (1/10, vol/vol) 1 ml of different culture media: BHI, BHI supplemented with 2% glucose, BHI supplemented with 0.2% sucrose or BHI supplemented with 1% sucrose. Finally, 200 μl of these inoculated media were seeded into two duplicated wells (~`2 × 10^6^ cells/ well). The E-plates were then connected to the RTCA-DP holder which had been previously placed for 2 hour into the incubator (37°C with 5% CO_2_). The biofilm formation was followed for 24 h, by recording the impedance signal (CI) every 10 min. The experiment was repeated with biological (independent bacterial cultures) replicates.

The staphylococcal biofilm formation was carried out as described previously [26]. Briefly, standardized grown cultures were diluted up to 10^7^ CFU/ml in fresh TSBG broth. Then, 200 μl of this suspension (~ 2x10^6^ cells/ well) were poured into the E-plates, which were connected to the RTCA-DP holder pre-warmed at 37°C. Biofilm formation was followed, as previously indicated, in independent biological replicates. In parallel, for comparison purposes *Staphylococcus* spp. biofilms were performed under the same conditions on other abiotic surfaces, such as polystyrene (TC Microwell 96U w/lid nunclon DSI plates, NUNC, Thermo Scientific, Madrid, Spain) and stainless steel (10×10×1 mm AISI 304 stainless steel coupons, Acerinox S.A., Madrid, Spain). Coupons were previously autoclaved at 121°C for 20 min, placed into a 24 flat-bottom microtiter plate and inoculated with 1 ml (~ 10^6^ cells/well) of staphylococcal cultures.

### Bacteriophage-based treatments on *Staphylococcus* spp. biofilm formation and removal

Additional experiments were carried out with the RTCA in order to check the effect of a bacteriophage and a bacteriophage-encoded endolysin on biofilm formation. In this case, 200 μl of *S*. *aureus* 15981 suspension (2 × 10^6^ cells/well) made in TSBG was supplemented with 0.15 μM of the endolysin LysH5, purified as described in a previous work [27]. Similarly, 200 μl of *S*. *epidermidis* F12 suspension made in TSBG was infected with the bacteriophage phi-IPLA7 (MOI 100) [28]. These bacterial suspensions, and their corresponding controls without treatment, were immediately added to E-plates and biofilm´s formation was monitored at 37°C for 24 h.

To assess biofilm elimination, 100 μl of *S*. *aureus* 15981 (10^6^ CFU/ml) in TSBG were added to each E-plate and biofilm growth was monitored for 8 h at 37°C. At this time, once the biofilms reached the stationary phase (CI constant), 100 μl of different LysH5 concentrations (from 0.05 to 2.88 μM, diluted in TSBG) were added; a control sample without LysH5 addition was also included as reference. The effect of LysH5 over preformed biofilms was monitored at 37°C during additional 6 h. This experimental procedure was repeated three times. The RTCA software 1.2.1 (ACEA Bioscience) was used for data normalization [29]: CI results obtained from each LysH5 concentration were referred to the CI obtained at the time of the bio-active addition and, afterwards, they were subtracted from the corresponding value of the reference sample.

### Biofilm detection and assessment

Crystal violet staining was used to determine the total biomass adhered to the gold-microelectrodes of the E-plate wells or to other abiotic surfaces. Culture medium supernatants were carefully removed and biofilms were washed twice with PBS buffer (137 mM NaCl, 2.7 mM KCl, 10 mM Na_2_HPO_4_ and 2 mM KH_2_PO_4_; pH 7.4), air-dried for 15 min at room temperature, then stained with crystal violet (0.1% wt/vol) and, finally, gently washed with tap water. *S*. *mutans* stained biofilms were photographed, whereas those of *Staphylococcus* spp. were de-stained with acetic acid (33%) and the absorbance of the supernatants was measured at 595 nm in a Microplate Benchmark Plus (BioRad, Hercules, CA) spectrophotometer.

Counts of *S*. *mutans* planktonic cultures were carried out after 24 h incubation in the four culture media used to test biofilm formation. For counting, serial dilutions were made in Ringer ¼ solution and plated on agar-BHI plates, which were incubated for 48 h under standard conditions. Counts of *Staphylococcus* spp. in biofilms were performed to determine the number of adhered cells that form the biofilm. For this purpose, wells were washed twice with PBS buffer and adhered cells were released by scratching twice with sterile swabs which were immersed in 9 ml of PBS buffer. The biofilm was further disaggregated by vigorous shaking for 1 min. Finally serial dilutions were plated onto TSA and incubated at 37°C for 24 h.

### Statistical analysis

The statistical analysis of the different measured parameters was performed with, at least, four data: two independent biological replicates each measured in duplicate. For this purpose, the statistical package IBM SPSS Statistics for Window Version 22.0 (IBM Corp., Armonk NY) was used to assess differences by means of one-way ANOVA tests. Additionally, when needed, the Duncan mean comparison test was used (p<0.05) to determine the differences among strains, culture media used and/or bacteriophage or LysH5 treatments. The legend of each figure or table shows the comparison performed. On the other hand, linear regression equations among different numeric parameters were calculated in order to obtain the coefficients of determination (R^2^) which show how well data fit to the regression line equations.

## Results

### The RTCA method monitored sucrose-dependent *S*. *mutans* biofilm formation

**Fig 2:**
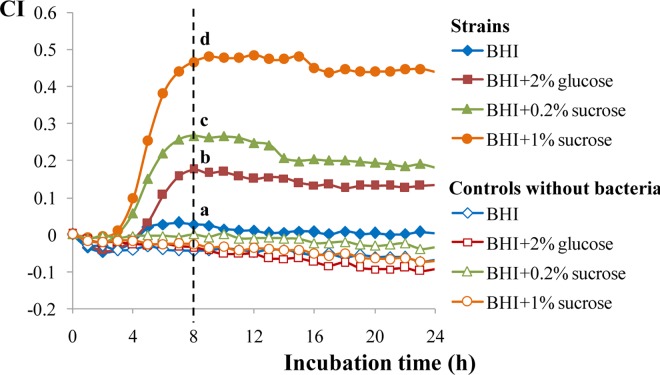
Variation in the cell index (CI) during *S*. *mutans* NCTC10449 biofilm formation, at 37°C under 5% CO_2_, depending on the carbon source added to the culture medium. At 8 h of incubation time, those values that have not a common letter are statistically different according to the Duncan mean comparison test (p<0.05). From this point of time, the percentage of coefficient of variation (100*SD/mean) of the data typically varied from 1% to 10%.

**Fig 3:**
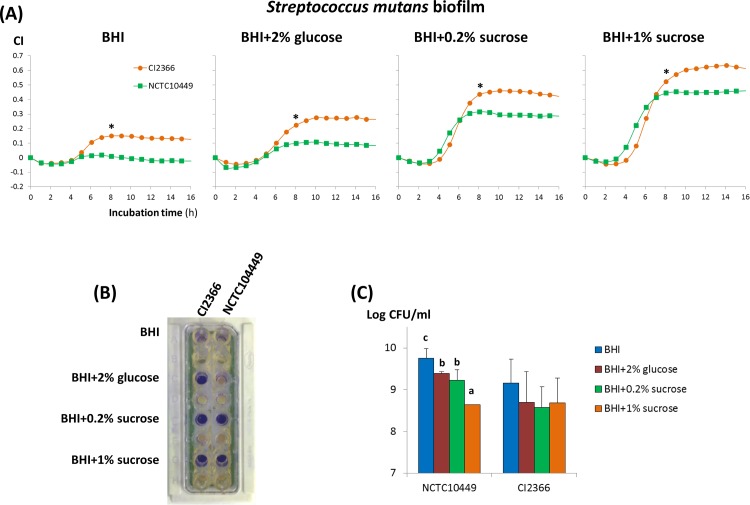
Variation in the cell index (CI) during biofilm formation, at 37°C under 5% CO_2_, of *S*. *mutans* NCTC10449 (type strain) and CI2366 (clinical isolate) depending on the carbon source added to the culture medium. At 8 h of incubation, the presence of the asterisk indicates that CI values were statistically different according to one-way ANOVA tests (p<0.05); the coefficient of variation (100*SD/mean) of data typically varied from 2% to 16% for strain CI2366, and from 1% to 30% for strain NCTC10449 (**A**). Crystal violet staining of the biofilms NCTC10449 and CI2366 formed in the E-plate (**B**). Counts (Log CFU/ml) of planktonic cultures of both strains in different culture media. Within each strain, the bars that do not share a common letter are statistically different according to ANOVA and Duncan mean comparison tests (p<0.05) (**C**).

The type strain *S*. *mutans* NCTC10449 was initially used to test the suitability of the RTCA technology to monitor the biofilm formation of this bacterium in real time. Since the occurrence of an oral biofilm in which *S*. *mutans* could be involved is directly correlated with the presence of sucrose in the environment, four BHI-based media were tested. Results obtained showed an increase in the CI signal in the media containing sucrose or high percentage of glucose, whereas in BHI, which is formulated by the commercial brand with 0.2% glucose, had no appreciable increase in the CI (Fig 2). The maximum CI values were reached after 8 h of incubation in all media, and the highest (p<0.05) value was obtained with 1% sucrose (CI around 0.5), followed by 0.2% sucrose and 2% glucose, which indicates that high biomass was adhered to the E-plate under the first condition. Of note it is the absence of signal interfering with the impedance measurement due to the ionic composition of the four culture media (Fig 2) which are based on BHI but having different concentrations of glucose or sucrose. The clinically isolated CI2366 strain was used to corroborate that the RTCA technology was detecting the formation of a biofilm. In this case, also a direct correlation between CI and presence of sucrose in the culture medium was denoted (Fig 3A). Besides, it seems that the clinical isolate was able to form stronger biofilms upon the gold-microelectrodes than the type strain since the CI index measured 8-h post-seeding was higher in all conditions (p<0.01) reaching a maximum value of 0.6. Crystal violet staining confirmed the presence of a biofilm in the corresponding inoculated wells, which seems to be weaker in the BHI medium without additional carbon source supply (Fig 3B). Finally, it is remarkable that the observed effect was not due to differences in the ability of the two strains to grow in the four culture media, given that the counts of planktonic cultures were similar, or even higher (p<0.05), in the absence of sucrose (Fig 3C).

### The RTCA method detected strain-dependent *Staphylococcus* spp. biofilm formation

**Fig 4:**
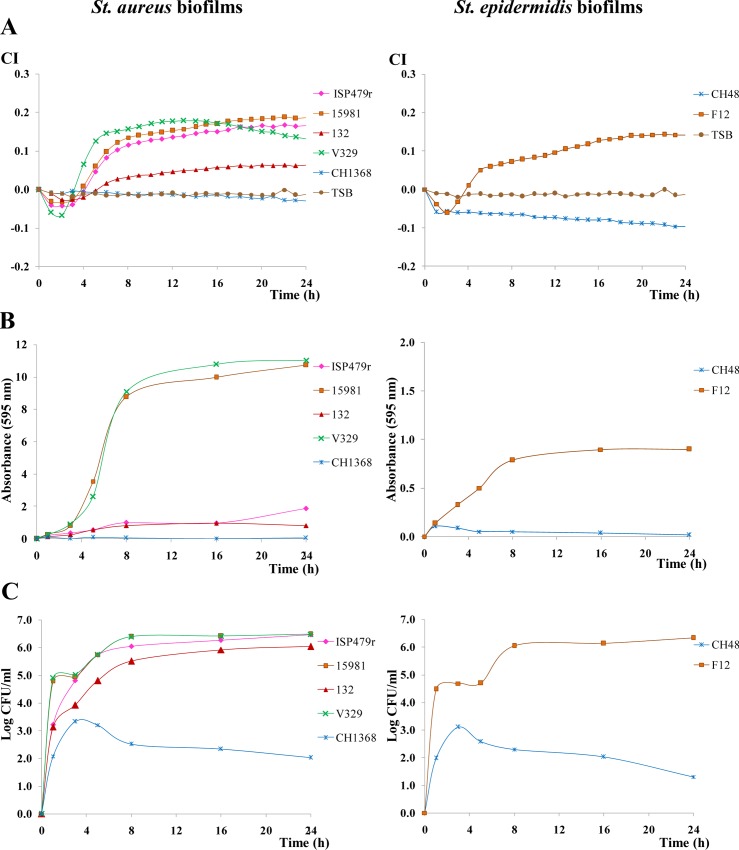
Variation in the cell index (CI) during biofilm formation at 37°C of different *S*. *aureus* and *S*. *epidermidis* biofilm producers (ISP479r, 15981, 132 or V320 and F12, respectively) and no-biofilm producers (CH1368 and CH48, respectively). TSB+0.25% glucose was the culture medium used in the experiment (**A**). Absorbance (595 nm) measured after crystal violet staining of samples collected at different times during the biofilm formation in E-plates of the strains under study (**B**). Counts (Log CFU/ml) of cells collected from the biofilms formed in the E-plates by the strains under study (**C**). Statistical differences among strains at three sampling points (8, 16 and 24 h) are collected in S1 Table, which also shows representative mean and SD values.

Two species of *Staphylococcus*, typically involved in biofilm production, were tested to further confirm the suitability of this impedance-based method to monitor formation of other bacterial biofilms. The four biofilm-producing *S*. *aureus* strains showed an increase in the CI signal which was not detected in the non-biofilm producing CH1368 strain (Fig 4A). Besides, statistical differences were detected among strains at different incubation times (S1 Table) and the maximum CI values reached varied between 0.06 and 0.19, depending on the strain considered. In this regard, the differences in the CI were not further correlated with the nature of the polymeric material forming the biofilm. In fact, the protein matrix producer V329 showed the highest CI for the first 15 h, decreasing afterwards, while the PIA/PNAG-polysaccharide producers ISP497r and 15981 reached higher CI from this time to the end of incubation period. However, the strain 132, also synthesizing PIA/PNAG, showed the lowest CI values throughout the incubation period (Fig 4A, S1 Table). Similar results were obtained using data of absorbance measured after crystal violet staining in all biofilms and they were also correlated with the strain regardless the type of matrix produced (Fig 4B, S1 Table). The bacterial counts of the *S*. *aureus* adhered to the gold-microelectrodes also showed statistical differences (Fig 4C, S1 Table); all biofilm-producing strains reached the maximum counts around 6.0 to 6.5 log units. Results obtained with the *S*. *epidermidis* model also confirm the capability of the RTCA technology to monitor this biofilm formation; CI values only increased in wells seeded with the biofilm-producing F12 strain, as well as crystal violet absorbance and the number of bacteria adhered to gold microelectrodes (Fig 4A, 4B and 4C, S1 Table).

It is truly remarkable that CI measurement was the only technique allowing the clear distinction among the four *S*. *aureus* strains. According to crystal violet staining the strains ISP479r and 132 could be both classified as weak biofilm producers given that no statistical differences were detected between them (S1 Table). Similarly, the stronger biofilm producers 15981 and V329 did not show any significant difference either with the staining method or with the counting technique. The CI determination allowed, therefore, a better classification of the strains based on their ability to adhere to the abiotic surface since the standard methods (bacterial viable counting and crystal violet staining) could probably be more influenced by the intrinsic characteristics of each strain, such as the nature of the polymeric material involved in adhesion or the bacterial growth rate, among other factors [30]. In this regard, the coefficients of determination (R^2^) obtained from linear regression equations comparing (two by two) data of CI, absorbance and bacterial counts, were calculated to correlate the three methods used for biofilm quantification purposes (Table 2). It is remarkable that only in the biofilm-producing strains, either from *S*. *aureus* or *S*. *epidermidis*, the R^2^ values were, in general, higher than 0.93, thus indicating a good fit between data. On the contrary, there was not linear relationship for the two non-biofilm producers in none of the three comparisons performed (Table 2).

**Table 2:** Linear regression coefficients of determination (R^2^) calculated from the parameters measured in *Staphylococcus* spp. strains biofilm producers and non-producers. At least five sampling points along the incubation time were used for the linear regression calculation (see in supplementary material “S1 Fig” the linear regression equations calculated for the biofilm producers).

			Linear regression coefficients (R^2^)*		
Species	Strain	Biofilm forming ability	CI vs. Abs	CI vs. Log CFU	Abs vs. Log CFU
*S*. *aureus*	15981	Biofilm producer	0.9860	0.9707	0.9529
	ISP479r	Biofilm producer	0.9558	0.9692	0.6908
	132	Biofilm producer	0.8828	0.9719	0.9849
	V329	Biofilm producer	0.9393	0.9605	0.9873
*S*. *epidermidis*	F12	Biofilm producer	0.9413	0.9521	0.9814
*S*. *aureus*	CH1368	No-biofilm producer	0.1128	0.5074	0.0116
*S*. *epidermidis*	CH48	No-biofilm producer	0.1895	0.6201	0.0171

* CI, cell index; Abs, absorbance; CFU, colony forming units.

It should be noticed that the use of RTCA technology implies that biofilm is formed upon gold surface which is not the standard material used for these studies. Therefore, in order to check whether this material could have influence on the results obtained, biofilms were also performed under the same conditions upon polystyrene and stainless steel surfaces. The absorbance after crystal violet staining was measured in biofilms formed upon the three materials. The linear regression coefficients R^2^ obtained with the five biofilm-producing strains showed good fit values, higher than 0.97 (S2 Fig) among the three materials tested. This indicates that the *S*. *aureus* and *S*. *epidermidis* strains studied were able to form similar biofilms on the three abiotic surfaces.

### Validation of RTCA method to monitor the inhibition of *Staphylococcus* spp. biofilm formation, and its removal, by bacteriophage and phage-derived proteins

**Fig 5:**
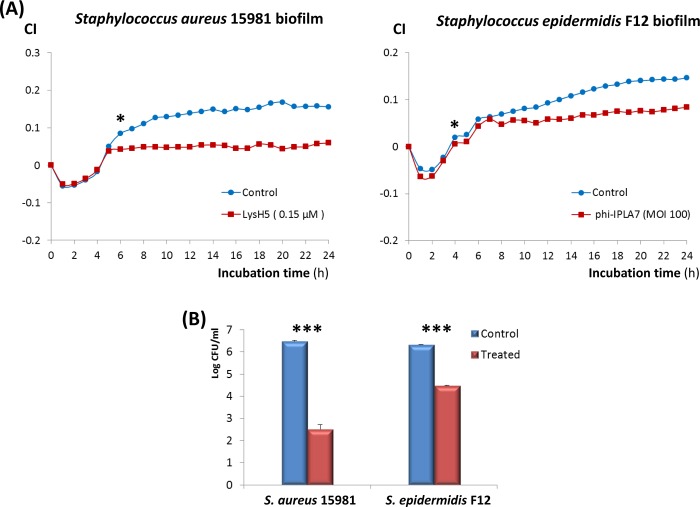
**Inhibition of the biofilm formation by *S*. *aureus* 15981 due to the addition of endolysin LysH5 (0.15** μ**M) to the culture medium TSBG, and by *S*. *epidermidis* F12 due to the addition of the bacteriophage phi-IPLA7 (MOI 100) to TSBG expressed as variation of the CI during biofilm treatment**; asterisks show the first incubation time after which two or more consecutive values of CI were statistically different (p<0.05), according to one-way ANOVA tests (S2 Table) **(A).** Counts (Log CFU/ml) of cells collected, after 24 h of treatment, from the biofilms formed in the E-plates; within each strain, the asterisks (p<0.001) show statistical differences according to ANOVA (**B**).

One of the most active areas of research in staphylococci biofilm studies is the screening of new bio-actives able to avoid the progression or to eliminate these microbial structures. Thus, we have applied the RTCA method to evaluate the capability of a staphylococcal bacteriophage and an endolysin to modify biofilm formation dynamics. *S*. *aureus* 15981 suspensions treated with 0.15 μM LysH5 immediately before seeding the E-plates were not able to reach CI values similar to the control sample (Fig 5A); the statistical differences between control and treated samples were detected around 6-h post-seeding (S2 Table). Similarly, the infection of *S*. *epidermidis* F12 with bacteriophage phiIPLA7 (MOI 100) also inhibited the biofilm formation and the statistical differences, with respect to the non-treated bacterial suspension, were first detected after 4 h of incubation (Fig 5A, S2 Table). After 24 h of incubation, counts of the remaining adhered cells also confirmed these results since they were reduced by 2 log-units in the wells treated with the endolysin and by 4 log units in the wells treated with the phage, respectively, compared with the untreated controls (Fig 5B).

**Fig 6:**
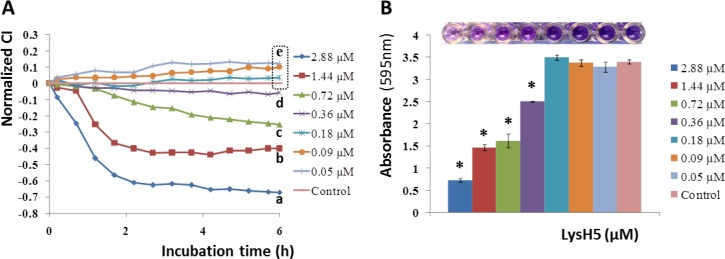
Removal of 8 h preformed *S*. *aureus* 15981 biofilms by endolysin LysH5 (added to TSBG from 0.05 to 2.88 μM) reported as variation of the normalized cell index (CI). At the final time, values having distinct letter are statistically different (p<0.05) according to the Duncan mean comparison test; the coefficient of variation (100*SD/mean) of these data typically varied from 5% to 38% (**A**). Absorbance (595 nm) measured after crystal violet staining of biofilms after 6 h of LysH5 treatment. Values with asterisks are statistically different (p<0.05) from the reference control (without LysH5 addition) according to one-way ANOVA tests. The photograph on the top shows the biofilms formed in the E-plates, in the presence of different concentrations of LysH5, stained with crystal violet (**B**).

The effect of LysH5 on 8 h preformed *S*. *aureus* 15981 biofilms was also tested using different concentrations of the endolysin. After 6 h of incubation, the values of the normalized CI (Fig 6A) showed that, in the range tested, 0.36 μM was the minimum effective dose that resulted statistically different (p<0.05) from the control sample (without LysH5). Lower doses were not able to eliminate the biofilms given that the normalized CI value did not differ from the control. At higher doses, the ability to detach the preformed staphylococcal biofilm was detected at shorter incubation periods. Indeed, the lowest normalized CI was achieved 2 h post-treatment in the presence of both 1.44 and 2.88 μM of LysH5; longer exposures to these concentrations did not improve the capability of LysH5 to remove the biofilm. These RTCA results were further validated by a final end-point method (after 6 h of incubation at 37°C), i.e. the crystal violet staining using both qualitative (photo) and quantitative (absorbance) measurements (Fig 6B).

Finally, it is worth noting the reproducible results obtained with the RTCA in our model bacteria, supported by the acceptable (lower than 38%) coefficient of variation (SD/mean, a measure of data dispersion) determined. Besides, another example illustrating the high reproducibility of the RTCA methodology has been provided by the strain *S*. *aureus* 15981 in three independent experiments; in all of them the maximum CI (around 0.18) was reached after 6–8 h of incubation (Fig 1, Fig 4 and Fig 5).

## Discussion

The ability of bacteria to adhere to different surfaces resulting in the formation of a biofilm seems to be a ubiquitous bacterial feature since it provides an advantage to resist adverse environmental conditions and facilitates cells dispersion to colonize different ecological niches [31]. As mentioned above, the microorganisms growing in biofilms have negative implications in health but also can affect different industrial processes such as, among others, food manufacturing [32]. The great number of biofilms implications worldwide has fueled the research in this field; in particular, the molecular mechanisms involved in biofilm formation, the characterization of biofilm structure, or the development of systems to control the biofilm formation or to facilitate its removal have been approached. For this purpose, one of the most frequently used *in vitro* biofilm model is the microtiter plate (MTP)-based system where biofilms grow on the bottom and the walls of microtiter-plate wells, or onto the surface of coupons placed on the bottom of wells. These biofilms are mostly visualized after crystal violet staining or using other labeling (e.g. fluorescence probes) approaches. This MTP method is a closed system that facilitates the screening of the potential biofilms producers in different nutrients and environmental conditions [33], as well as testing the sensitivity of biofilm-forming bacteria to different antimicrobials [34]. However, processing of biofilms to calculate biomass and adhered cells in this MTP-based system is a time consuming, end-point and label-dependent method; for this reason, alternative devises are currently under development to continuously monitor biofilm formation, such as those using time-lapsed image recording [35,36], methods for mass measurement by using piezoelectric tuning fork [37], or those based on electrical impedance spectroscopy measurements [38,39]. In the current work we have analyzed the suitability of the xCELLigence (RTCA) equipment, a non-invasive, label-free, real time monitoring system based on impedance (CI) recording, to follow biofilm formation. This technology was previously used to measure biofilm formation in one strain of *S*. *aureus* [40] and, more recently, to follow biofilm disruption after antibiotic and selenium-nanoparticles treatments in two strains of the same species [41]. However, in our study we have validated this methodology using nine strains (including non-biofilm producing controls) from three model species, in comparison with two conventional methodologies and two abiotic surfaces.

In our study the results obtained with *S*. *mutans* clearly showed that the RTCA equipment was able to monitor the formation of biofilms upon the gold-microelectrodes of the E-plates, which occurred when sucrose was present in the culture medium especially at higher concentrations (1%). It has been reported that sucrose is a cariogenic sugar, favoring dental biofilm formation, since it is used as fermentable substrate by pro-cariogenic members of oral microbiota, promotes higher enamel mineral loss than other sugars, and also serves as substrate for streptococcal GTFs involved in the synthesis of glucans [42]. Additionally, differences on sucrose-dependent adherence were also monitored between the clinical isolate (strain CI2366) and the type strain (NCTC10449); this fact underlines that the impedance method was able to distinguish different biofilm formation capabilities, which it is known to be a strain-dependent trait in *S*. *mutans* [43,44]. Regarding staphylococcal biofilms, the impedance CI increased throughout time only in those positive biofilm-forming strains (15981, ISP479r, 132, V329 and F12), also in a strain dependent manner regardless composition of the biofilm matrix. With the only exception of *S*. *aureus* V329 strain, the highest CI values were detected at 24 h being higher than those observed previously for *S*. *aureus* ATCC6538 [40]. The strain V329 showed a continuous CI decrease, after the maximum reached at 16 h, which can be explained by dispersal/detachment phenomena that occur in staphylococcal biofilms during the maturation process [45]. These observations suggest that the RTCA equipment was sensitive enough to detect differences among biofilm producing conditions and biofilm producer strains. As expected, at defined incubation times higher absorbance values (as expression of biomass production) were detected in biofilm-producing strains after crystal violet staining than in non-producers, as well as higher counts of adhered cells. Remarkably, a strong correlation was obtained (R^2^≥ 0.93) when CI index was compared either with absorbance values or adhered cells counts in biofilm-producing strains. This was also observed (R^2^≥ 0.98–0.99) in absorbance values when compared biofilm developed on the gold electrodes (hydrophilic) with those developed either on polystyrene (hydrophobic) wells or stainless steel coupons (hydrophilic). This is not a surprising result since da Silva Meira and co-workers [46] observed similar capability of staphylococcal isolates to adhere and form biofilms on hydrophilic or hydrophobic surfaces. Therefore, the good correlation obtained among all parameters tested using either standard methods or the RTCA technology, indicates that the latter is a suitable alternative to study biofilm formation in real time. The CI index values reached during bacterial biofilm formation were about 10-times lower than those typically detected for adherent eukaryotic cells [25]; this could be related to the different size between prokaryotic and eukaryotic cells.

The usual tolerance of biofilms to antimicrobial agents (antibiotics and disinfectants) has promoted the emergence of alternative strategies to prevent the biofilm formation or removal of preformed biofilms, such as the use of bacteriophages [47] and phage-encoded enzymes [26,48]. Regarding this, the previously characterized bacteriophage phi-IPLA7 [28,49] and the endolysin LysH5 [26,27] were used as antimicrobials to test their capability to inhibit staphylococcal biofilm formation using the RTCA equipment. After 4–6 h of incubation, this technology recorded significant differences in the CI values obtained for *S*. *aureus* 15981 and *S*. *epidermidis* F12 in the presence of these antimicrobials, with respect to those in the control samples, indicating that biofilm formation was prevented. Moreover, the usefulness of this technique to monitor the efficacy of the endolysin LysH5 over a preformed staphylococcal biofilm was demonstrated, and the most effective dose for biofilm removal was determined. Previous reports have also showed the inhibition in biofilm formation by bacteriophages [50–52] and the successful removal of these structures by endolysins [26,53,54] as revealed by crystal violet staining and viable cell counts. However, as far as we know, this is the first time that an impedance-based method has been used to show the ability of bacteriophages and phage lytic enzymes to reduce or to remove the biofilms formed by staphylococcal strains.

## Conclusion

Overall, results obtained in this work support that the impedance-based RTCA monitoring is a fast, reliable, and no-time consuming method than can be easily used, among other applications, to perform screening of strains able to form biofilms, as well as to search for bio-actives that interfere the biofilm formation, or remove the preformed ones, upon abiotic (gold) surface; this methodology also allows to determine the antimicrobial effective concentration. Additional advantages, with respect to the standard methods applied to biofilm research, are that it is not an end-point method since continuously monitor the biofilm formation and it is a label-free technique given that impedance curves are denoted in real-time without need of staining procedures. Regarding disadvantages, it is worth mentioning the higher cost of the consumables (E-plates) of the RTCA in comparison with those of the traditional MTP-based systems. Besides, the impedance-based technologies are highly dependent on the different ionic strength and concentration of multi-valent ions of the electrically conductive solutions (buffer or culture medium) that could affect biofilm formation and may also influence impedance signals. Therefore, it is worth noting that this technology could not be suitable for all type of bacteria, but only for those able to form biofilms upon gold material and under ion-strength conditions that do not affect the basal impedance signal. Thus, other model bacteria should be tested in order to find its range of applicability.

## Supporting Information

S1 Fig Linear regression equations and coefficients of determination (R^2^) comparing, two by two, the CI, absorbance and counts determined for each biofilm-producer staphylococcal strain. At least five sampling points, along the incubation time, were used for the linear regression calculation.
(PDF)

S2 Fig Linear regression equations and coefficients of determination (R^2^) calculated from the absorbance data of S*taphylococcus* spp. biofilms formed in three abiotic surfaces, compared two by two. Six sampling points, along the incubation time, were used for the linear regression calculation.
(PDF)

S1 Table Statistical analysis of biofilm-related parameters measured at three incubation times. One-way ANOVA tests were performed to establish differences among strains belonging to the same species (*** p<0.001). When needed, mean comparison Duncan tests (p<0.05) were carried out to assign differences among the strains; then, values that do not share a common superscript letter are different.
(PDF)

S2 Table One-way ANOVAs to assess differences between control and treated (endolysin LysH5 or bacteriophage phi-IPLA007) samples during biofilm formation. Differences were considered stable when two or more consecutive p-values were lower than 0.05.
(PDF)

## References

[1] Flemming HC, Wingender J (2010) The biofilm matrix. Nat Rev Microbiol 8: 623–633. 10.1038/nrmicro2415 20676145

[2] Solano C, Echeverz M, Lasa I (2014) Biofilm dispersion and quorum sensing. Curr Opin Microbiol 18: 96–104. 10.1016/j.mib.2014.02.008 24657330

[3] Romling U, Balsalobre C (2012) Biofilm infections, their resilience to therapy and innovative treatment strategies. J Intern Med 272: 541–561. 10.1111/joim.12004 23025745

[4] Teschler JK, Zamorano-Sanchez D, Utada AS, Warner CJ, Wong GC, Linington RG, et al (2015) Living in the matrix: assembly and control of *Vibrio cholerae* biofilms. Nat Rev Microbiol 13: 255–268. 10.1038/nrmicro3433 25895940 PMC4437738

[5] Costerton JW, Cheng KJ, Geesey GG, Ladd TI, Nickel JC, Dasgupta M, et al (1987) Bacterial biofilms in nature and disease. Annu Rev Microbiol 41: 435–464. 10.1146/annurev.micro.41.1.435 3318676

[6] Donlan RM, Costerton JW (2002) Biofilms: survival mechanisms of clinically relevant microorganisms. Clin Microbiol Rev 15: 167–193. 10.1128/cmr.15.2.167-193.2002 11932229 PMC118068

[7] Singh R, Ray P, Das A, Sharma M (2010) Penetration of antibiotics through *Staphylococcus aureus* and *Staphylococcus epidermidis* biofilms. J Antimicrob Chemother 65: 1955–1958. 10.1093/jac/dkq257 20615927

[8] Hoiby N, Bjarnsholt T, Givskov M, Molin S, Ciofu O (2010) Antibiotic resistance of bacterial biofilms. Int J Antimicrob Agents 35: 322–332. 10.1016/j.ijantimicag.2009.12.011 20149602

[9] Romling U, Kjelleberg S, Normark S, Nyman L, Uhlin BE, Åkerlund B (2014) Microbial biofilm formation: a need to act. J Intern Med 276: 98–110. 10.1111/joim.12242 24796496

[10] Otto M (2008) Staphylococcal biofilms. Curr Top Microbiol Immunol 322: 207–228. 18453278 10.1007/978-3-540-75418-3_10 PMC2777538

[11] Kropec A, Maira-Litran T, Jefferson KK, Grout M, Cramton SE, Götz F, et al (2005) Poly-N-acetylglucosamine production in *Staphylococcus aureus* is essential for virulence in murine models of systemic infection. Infect Immun 73: 6868–6876. 10.1128/IAI.73.10.6868-6876.2005 16177366 PMC1230935

[12] Shiro H, Muller E, Gutierrez N, Boisot S, Grout M, Tosteson TD, et al (1994) Transposon mutants of *Staphylococcus epidermidis* deficient in elaboration of capsular polysaccharide/adhesin and slime are virulent in a rabbit model of endocarditis. J Infect Dis 169: 1042–1049. 10.1093/infdis/169.5.1042 8169389

[13] Cucarella C, Solano C, Valle J, Amorena B, Lasa I, Penadés JR (2001) Bap, a *Staphylococcus aureus* surface protein involved in biofilm formation. J Bacteriol 183: 2888–2896. 10.1128/jb.183.9.2888-2896.2001 11292810 PMC99507

[14] Corrigan RM, Rigby D, Handley P, Foster TJ (2007) The role of *Staphylococcus aureus* surface protein SasG in adherence and biofilm formation. Microbiology 153: 2435–2446. 10.1099/mic.0.2007/006676-0 17660408

[15] Izano EA, Amarante MA, Kher WB, Kaplan JB (2008) Differential roles of poly-N-acetylglucosamine surface polysaccharide and extracellular DNA in *Staphylococcus aureus* and *Staphylococcus epidermidis* biofilms. Appl Environ Microbiol 74: 470–476. 10.1128/AEM.02073-07 18039822 PMC2223269

[16] Simón-Soro A, Mira A (2015) Solving the etiology of dental caries. Trends Microbiol 23: 76–82. 10.1016/j.tim.2014.10.010 25435135

[17] Kolenbrander PE, Palmer RJ Jr., Periasamy S, Jakubovics NS (2010) Oral multispecies biofilm development and the key role of cell-cell distance. Nat Rev Microbiol 8: 471–480. 10.1038/nrmicro2381 20514044

[18] Mashima I, Nakazawa F (2015) Interaction between *Streptococcus* spp. and *Veillonella tobetsuensis* in the early stages of oral biofilm formation. J Bacteriol 197: 2104–2111. 10.1128/jb.02512-14 PMC4455269 25917902

[19] Koo H, Xiao J, Klein MI, Jeon JG (2010) Exopolysaccharides produced by *Streptococcus mutans* glucosyltransferases modulate the establishment of microcolonies within multispecies biofilms. J Bacteriol 192: 3024–3032. 10.1128/JB.01649-09 20233920 PMC2901689

[20] Jeon JG, Rosalen PL, Falsetta ML, Koo H (2011) Natural products in caries research: current (limited) knowledge, challenges and future perspective. Caries Res 45: 243–263. 10.1159/000327250 21576957 PMC3104868

[21] Soderling EM, Ekman TC, Taipale TJ (2008) Growth inhibition of *Streptococcus mutans* with low xylitol concentrations. Curr Microbiol 56: 382–385. 10.1007/s00284-007-9076-6 18176823

[22] Valle J, Toledo-Arana A, Berasain C, Ghigo JM, Amorena B, Penadés JR (2003) SarA and not sigmaB is essential for biofilm development by *Staphylococcus aureus*. Mol Microbiol 48: 1075–1087. 10.1046/j.1365-2958.2003.03493.x 12753197

[23] Vergara-Irigaray M, Valle J, Merino N, Latasa C, Garcia B, Ruiz de los Mozos I, et al (2009) Relevant role of fibronectin-binding proteins in *Staphylococcus aureus* biofilm-associated foreign-body infections. Infect Immun 77: 3978–3991. 10.1128/IAI.00616-09 19581398 PMC2738049

[24] Delgado S, Arroyo R, Jimenez E, Marin ML, del Campo R, Fernández L, et al (2009) *Staphylococcus epidermidis* strains isolated from breast milk of women suffering infectious mastitis: potential virulence traits and resistance to antibiotics. BMC Microbiol 9: 82 10.1186/1471-2180-9-82 19422689 PMC2685400

[25] Hidalgo-Cantabrana C, Kekkonen R, de los Reyes-Gavilán CG, Salminen S, Korpela R, Gueimonde M, et al (2014) Effect of bacteria used in food industry on the proliferation and cytokine production of epithelial intestinal cellular lines. J Funct Foods 6: 348–355. 10.1016/j.jff.2013.11.001

[26] Gutiérrez D, Ruas-Madiedo P, Martínez B, Rodríguez A, García P (2014) Effective removal of staphylococcal biofilms by the endolysin LysH5. PLoS One 9: e107307 10.1371/journal.pone.0107307 25203125 PMC4159335

[27] Obeso JM, Martínez B, Rodríguez A, García P (2008) Lytic activity of the recombinant staphylococcal bacteriophage PhiH5 endolysin active against *Staphylococcus aureus* in milk. Int J Food Microbiol 128: 212–218. 10.1016/j.ijfoodmicro.2008.08.010 18809219

[28] Gutiérrez D, Martínez B, Rodríguez A, García P (2012) Genomic characterization of two *Staphylococcus epidermidis* bacteriophages with anti-biofilm potential. BMC Genomics 13: 228 10.1186/1471-2164-13-228 22681775 PMC3505474

[29] Valdés L, Gueimonde M, Ruas-Madiedo P (2015) Monitoring in real time the cytotoxic effect of *Clostridium difficile* upon the intestinal epithelial cell line HT29. J Microbiol Meth 119: 66–73. 10.1016/j.mimet.2015.09.022 26436983

[30] Peeters E, Nelis HJ, Coenye T (2008) Comparison of multiple methods for quantification of microbial biofilms grown in microtiter plates, J Microbiol Meth 72: 157–165. 10.1016/j.mimet.2007.11.010 18155789

[31] Hall-Stoodley L, Stoodley P, Kathju S, Hoiby N, Moser C, Costerton JW, et al (2012) Towards diagnostic guidelines for biofilm-associated infections. FEMS Immunol Med Microbiol 65: 127–145. 10.1111/j.1574-695X.2012.00968.x 22469292

[32] Whitehead KA, Verran J (2015) Formation, architecture and functionality of microbial biofilms in the food industry. Curr Opin Food Sci 2: 84–91. 10.1016/j.cofs.2015.02.003

[33] Fernández-Ramírez MD, Smid EJ, Abee T, Nierop-Groot MN (2015) Characterisation of biofilms formed by *Lactobacillus plantarum* WCFS1 and food spoilage isolates. Int J Food Microbiol 207: 23–29. 10.1016/j.ijfoodmicro.2015.04.030 25965141

[34] Coenye T, Nelis HJ (2010) *In vitro* and *in vivo* model systems to study microbial biofilm formation. J Microbiol Methods 83: 89–105. 10.1016/j.mimet.2010.08.018 20816706

[35] Navarro G, Cheng AT, Peach KC, Bray WM, Bernan VS, Yildiz FH, et al (2014) Image-based 384-well high-throughput screening method for the discovery of skyllamycins A to C as biofilm inhibitors and inducers of biofilm detachment in *Pseudomonas aeruginosa*. Antimicrob Agents Chemother 58: 1092–1099. 10.1128/AAC.01781-13 24295976 PMC3910817

[36] Tremblay YD, Vogeleer P, Jacques M, Harel J (2015) High-throughput microfluidic method to study biofilm formation and host-pathogen interactions in pathogenic *Escherichia coli*. Appl Environ Microbiol 81: 2827–2840. 10.1128/AEM.04208-14 25681176 PMC4375333

[37] Gula G, Waszczuk K, Olszak T, Majewska J, Sarowska J, Gotszalk T, et al (2012) Piezoelectric tuning fork based mass measurement method as a novel tool for determination of antibiotic activity on bacterial biofilm. SensorActuat B- Chem 175: 34–39. 10.1016/j.snb.2011.11.044

[38] Dheilly A, Linossier I, Darchen A, Hadjiev D, Corbel C, Alonso V (2008) Monitoring of microbial adhesion and biofilm growth using electrochemical impedancemetry. Appl Microbiol Biotechnol 79: 157–164. 10.1007/s00253-008-1404-7 18330564

[39] Estrada-Leypon O, Moya A, Guimera A, Gabriel G, Agut M, Sanchez B, et al (2015) Simultaneous monitoring of *Staphylococcus aureus* growth in a multi-parametric microfluidic platform using microscopy and impedance spectroscopy. Bioelectrochem 105: 56–64. 10.1016/j.bioelechem.2015.05.006 26004850

[40] Junka AF, Janczura A, Smutnicka D, Maczynska B, Secewicz A, Nowicka J, et al (2012) Use of the real time xCelligence system for purposes of medical microbiology. Pol J Microbiol 61: 191–197. 29334048

[41] Cihalova K, Chudobova D, Michalek P, Moulick A, Guran R, Kopel P, et al (2015) *Staphylococcus aureus* and MRSA growth and biofilm formation after treatment with antibiotics and SeNPs. Int J Mol Sci 16: 24656–24672. 10.3390/ijms161024656 26501270 PMC4632770

[42] Paes-Leme AF, Koo H, Bellato CM, Bedi G, Cury JA (2006) The role of sucrose in cariogenic dental biofilm formation-new insight. J Dent Res 85: 878–887. 10.1177/154405910608501002 16998125 PMC2257872

[43] Zhao W, Li W, Lin J, Chen Z, Yu D (2014) Effect of sucrose concentration on sucrose-dependent adhesion and glucosyltransferase expression of *S*. *mutans* in children with severe early-childhood caries (S-ECC). Nutrients 6: 3572–3586. 10.3390/nu6093572 25207825 PMC4179176

[44] Wang Y, Lee SM, Dykes GA (2015) Growth in the presence of sucrose may decrease attachment of some oral bacteria to abiotic surfaces. Ann Microbiol 65: 1159–1163. 10.1007/s13213-014-0883-2

[45] Boles BR, Horswill AR (2011) Staphylococcal biofilm disassembly. Trends Microbiol 19: 449–455. 10.1016/j.tim.2011.06.004 21784640 PMC3164736

[46] da Silva Meira QG, de Medeiros Barbosa I, Alves Aguiar Athayde AJ, de Siqueira-Júnior JP, de Souza EL (2012) Influence of temperature and surface kind on biofilm formation by *Staphylococcus aureus* from food-contact surfaces and sensitivity to sanitizers. Food Control 25: 469–475. 10.1016/j.foodcont.2011.11.030 PMC4059327 24948915

[47] Donlan RM (2009) Preventing biofilms of clinically relevant organisms using bacteriophage. Trends Microbiol 17: 66–72. 10.1016/j.tim.2008.11.002 19162482

[48] Rodríguez-Rubio L, Gutiérrez D, Donovan DM, Martínez B, Rodríguez A, García (2015) Phage lytic proteins: biotechnological applications beyond clinical antimicrobials. Crit Rev Biotechnol: 1–11. 10.3109/07388551.2014.993587 25603721

[49] Gutiérrez D, Martínez B, Rodríguez A, García P (2010) Isolation and characterization of bacteriophages infecting *Staphylococcus epidermidis*. Curr Microbiol 61: 601–608. 10.1007/s00284-010-9659-5 20449591

[50] Fu W, Forster T, Mayer O, Curtin JJ, Lehman SM, Donlan RM (2010) Bacteriophage cocktail for the prevention of biofilm formation by *Pseudomonas aeruginosa* on catheters in an in vitro model system. Antimicrob Agents Chemother 54: 397–404. 10.1128/AAC.00669-09 19822702 PMC2798481

[51] Kelly D, McAuliffe O, Ross RP, Coffey A (2012) Prevention of *Staphylococcus aureus* biofilm formation and reduction in established biofilm density using a combination of phage K and modified derivatives. Lett Appl Microbiol 54: 286–291. 10.1111/j.1472-765X.2012.03205.x 22251270

[52] Knezevic P, Obreht D, Curcin S, Petrusic M, Aleksic V, Kostanjsek R, et al (2011) Phages of *Pseudomonas aeruginosa*: response to environmental factors and in vitro ability to inhibit bacterial growth and biofilm formation. J Appl Microbiol 111: 245–254. 10.1111/j.1365-2672.2011.05043.x 21554503

[53] Fenton M, Keary R, McAuliffe O, Ross RP, O'Mahony J, Coffey A (2013) Bacteriophage-derived peptidase CHAP(K) eliminates and prevents staphylococcal biofilms. Int J Microbiol 2013: 625341 10.1155/2013/625341 23431312 PMC3574654

[54] Meng X, Shi Y, Ji W, Meng X, Zhang J, Wang H, et al (2011) Application of a bacteriophage lysin to disrupt biofilms formed by the animal pathogen *Streptococcus suis*. Appl Environ Microbiol 77: 8272–8279. 10.1128/AEM.05151-11 21984241 PMC3233067

