# Supplementary material for: Monitoring in Real Time the Formation and Removal of Biofilms from Clinical Related Pathogens Using an Impedance-Based Technology
Source: PLoS One. 2016 Oct 3;11(10):e0163966. doi: 10.1371/journal.pone.0163966 (PMC5047529; doi:10.1371/journal.pone.0163966)

S. aureus

Time (h)	CI	Ab	Log CFU
1	-0.0314	0.29	4.79
3	-0.0274	0.81	4.94
5	0.0596	3.56	5.75
8	0.1349	8.78	6.41
16	0.1705	9.99	6.43
24	0.1865	10.75	6.49

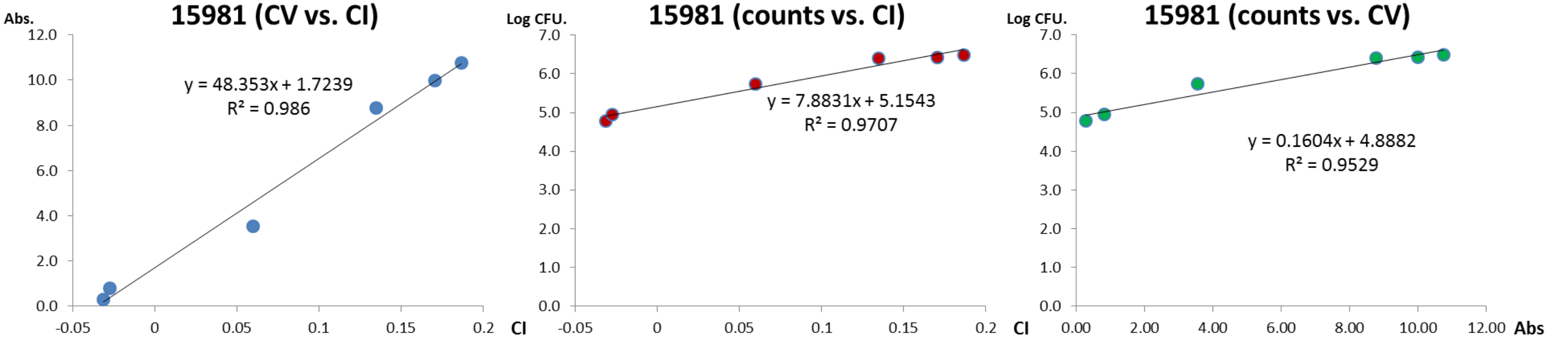

Time (h)	CI	Ab	Log CFU
1	-0.0418	0.22	3.22
3	-0.0390	0.35	4.81
5	0.0455	0.55	5.74
8	0.1154	0.98	6.06
16	0.1506	0.98	6.27
24	0.1665	1.87	6.48

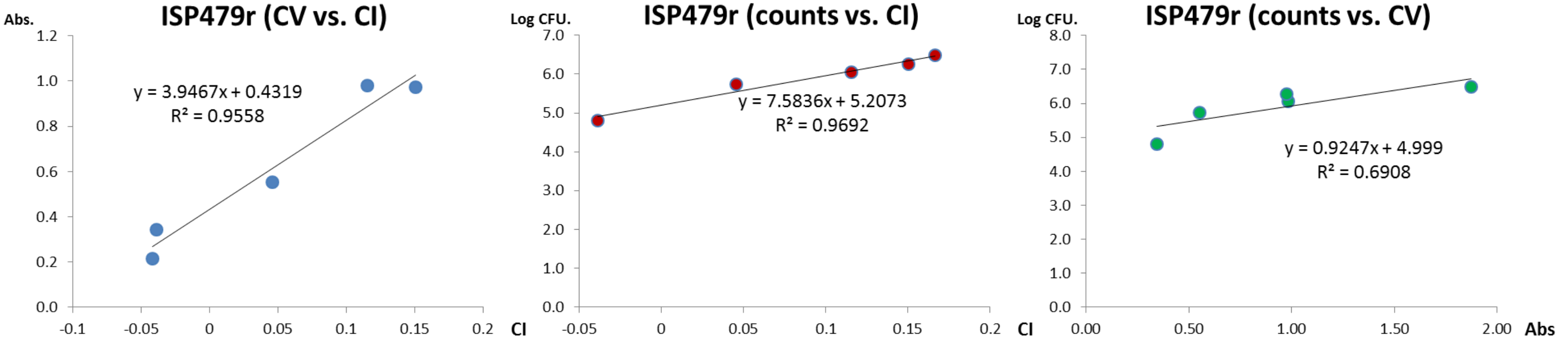

Time (h)	CI	Ab	Log CFU
1	-0.0105	0.11	3.14
3	-0.0263	0.25	3.92
5	-0.0027	0.54	4.81
8	0.0313	0.80	5.51
16	0.0575	0.96	5.93
24	0.0637	0.81	6.05

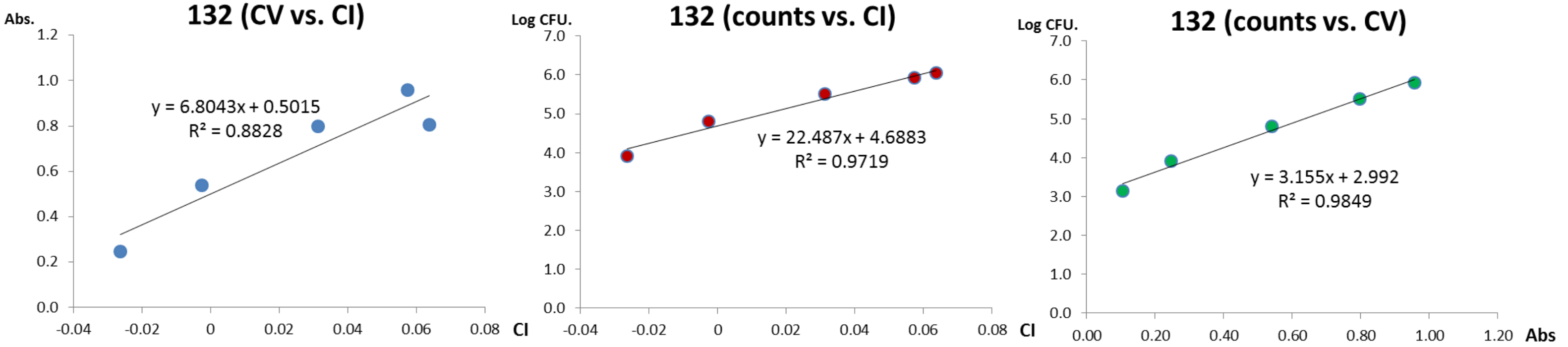

Time (h)	CI	Ab	Log CFU
1	-0.0599	0.24	4.91
3	-0.0109	0.92	5.03
5	0.1251	2.61	5.75
8	0.1570	9.09	6.41
16	0.1719	10.79	6.43
24	0.1320	11.04	6.49

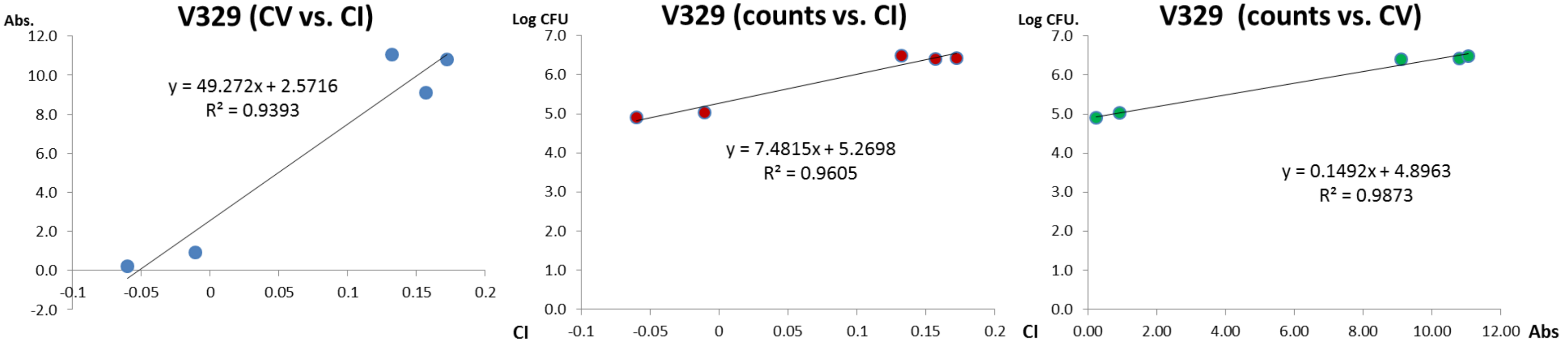

S. epidermidis

Time (h)	CI	Ab	Log CFU
1	-0.0380	0.13	4.48
3	-0.0318	0.32	4.69
5	0.0507	0.50	4.70
8	0.0723	0.79	6.05
16	0.1269	0.90	6.14
24	0.1423	0.90	6.34

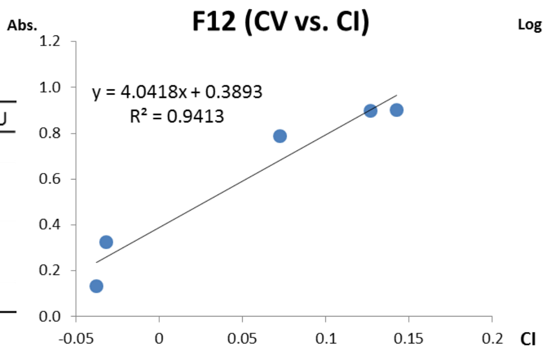

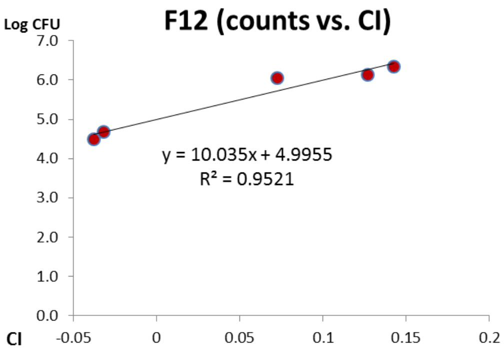

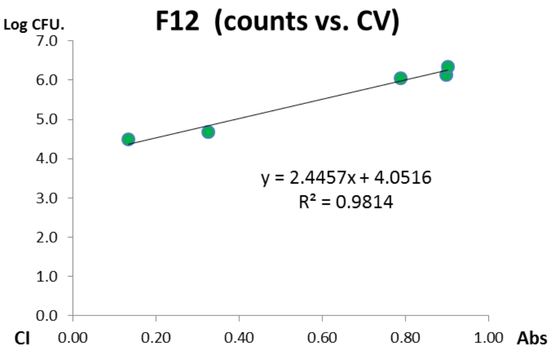

Supplement: S1 Fig — At least five sampling points, along the incubation time, were used for the linear regression calculation. (PDF) [file pone.0163966.s001.pdf]
