# Supplementary material for: Monitoring in Real Time the Formation and Removal of Biofilms from Clinical Related Pathogens Using an Impedance-Based Technology
Source: PLoS One. 2016 Oct 3;11(10):e0163966. doi: 10.1371/journal.pone.0163966 (PMC5047529; doi:10.1371/journal.pone.0163966)

S. aureus

Strain 15981

Time (h)	Gold	Polyst.	S. Steel
1	0.290	0.350	0.291
3	0.809	0.635	0.729
5	3.557	3.566	3.320
8	8.777	8.62	8.442
16	9.988	10.327	9.686
24	10.750	10.370	10.633

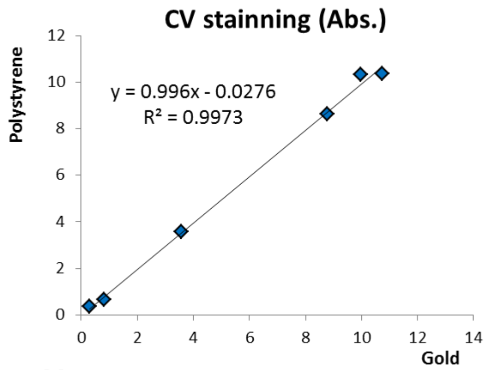

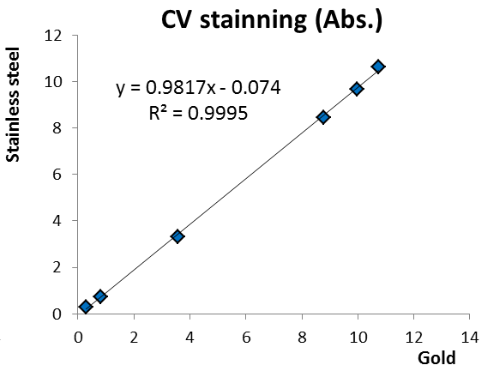

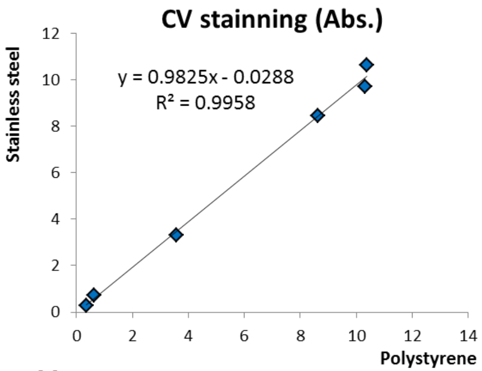

Strain ISP479r

Time (h)	Gold	Polyst.	S. Steel
1	0.217	0.138	0.302
3	0.345	0.382	0.397
5	0.552	0.557	0.573
8	0.982	0.927	0.953
16	0.975	1.078	1.109
24	1.873	1.823	

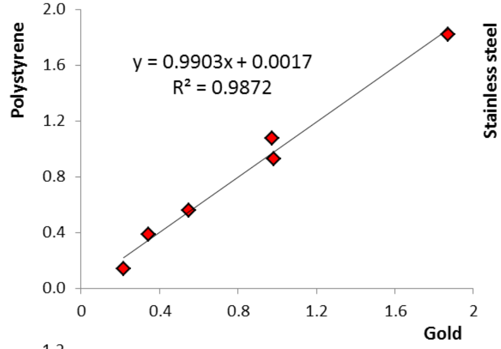

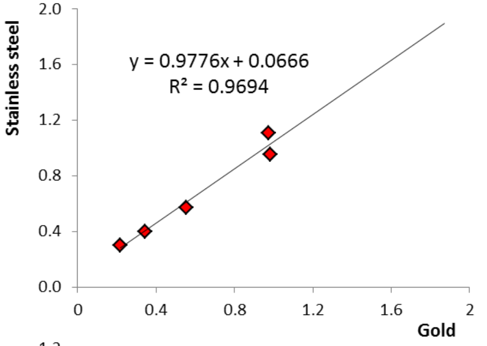

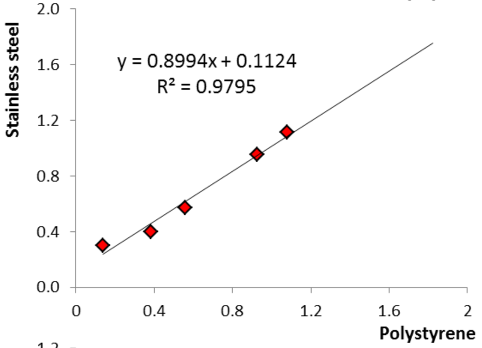

Strain 132

Time (h)	Gold	Polyst.	S. Steel
1	0.106	0.101	0.163
3	0.247	0.287	0.273
5	0.540	0.503	0.558
8	0.798	0.786	0.788
16	0.958	0.977	1.039
24	0.806	0.925	0.888

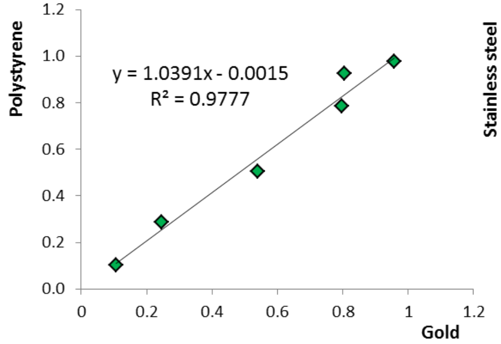

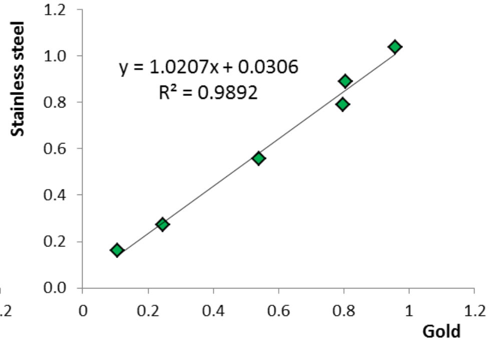

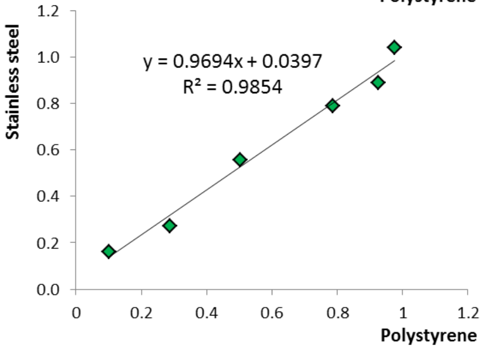

Strain V329

Time (h)	Gold	Polyst.	S. Steel
1	0.240	0.227	0.274
3	0.920	0.885	0.918
5	2.610	2.300	2.486
8	9.090	9.257	9.720
16	10.790	10.290	10.367
24	11.040	10.950	10.820

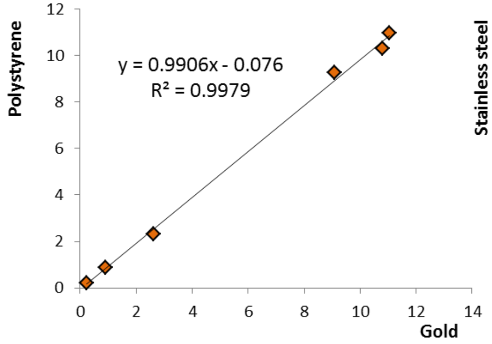

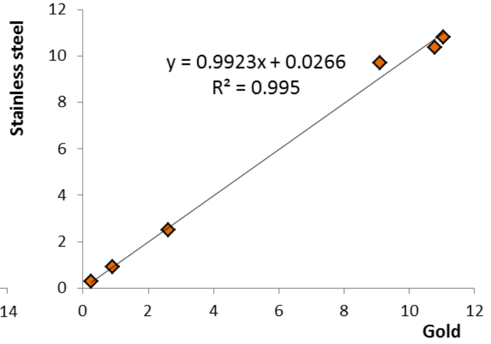

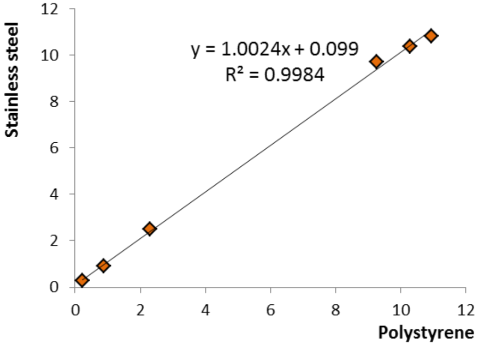

S. epidermidis

Strain F12

Time (h)	Gold	Polyst.	S. Steel
1	0.134	0.207	0.230
3	0.325	0.339	0.349
5	0.500	0.527	0.490
8	0.788	0.795	0.726
16	0.898	0.881	0.835
24	0.901	0.914	0.856

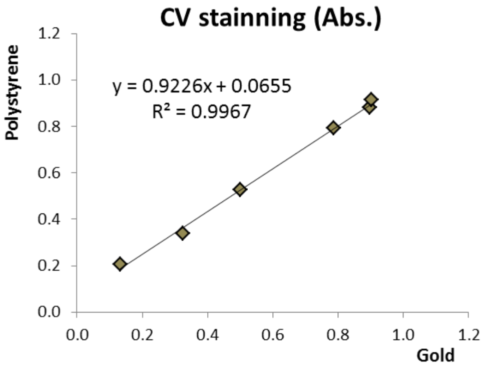

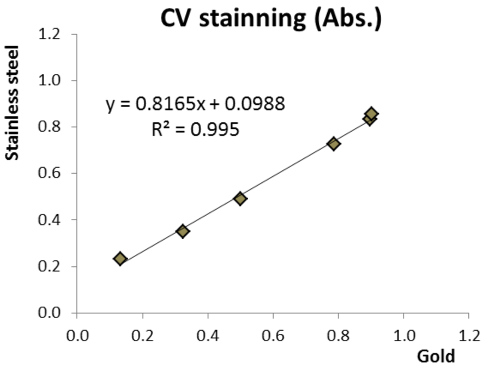

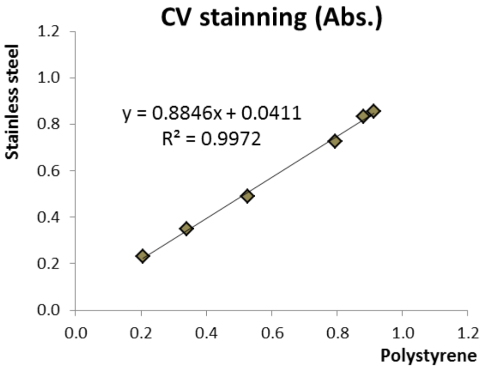

Supplement: S2 Fig — Six sampling points, along the incubation time, were used for the linear regression calculation. (PDF) [file pone.0163966.s002.pdf]
