# Supplementary material for: Monitoring in Real Time the Formation and Removal of Biofilms from Clinical Related Pathogens Using an Impedance-Based Technology
Source: PLoS One. 2016 Oct 3;11(10):e0163966. doi: 10.1371/journal.pone.0163966 (PMC5047529; doi:10.1371/journal.pone.0163966)
Supplement: S1 Table — One-way ANOVA tests were performed to establish differences among strains belonging to the same species (*** p<0.001). When needed, mean comparison Duncan tests (p<0.05) were carried out to assign differences among the strains; then, values that do not share a common superscript letter are different. (PDF) [file pone.0163966.s003.pdf]

			Mean ± SD		
**Time**	**Species**	**Strain**	**CI**	**Absorbance**	**Log CFU/ml**
8 h	*S. aureus*	ISP479r	0.115±0.006 [c]	0.982±0.004 [b]	6.05±0.06 [c]
		15981	0.135±0.013 [d]	8.777±0.181 [c]	6.40±0.09 [d]
		132	0.031±0.003 [b]	0.798±0.012 [b]	5.51±0.01 [b]
		V329	0.157±0.003 [e]	9.091±0.493 [c]	6.41±0.11 [d]
		CH1368	0.012±0.000 [a]	0.044±0.019 [a]	2.51±0.01 [a]
			***	***	***
	S. epidermidis	F12	0.072±0.003	0.788±0.002	6.05±0.04
		CH48	-0.066±0.001	0.051±0.015	2.30±0.02
			***	***	***
16 h	*S. aureus*	ISP479r	0.151±0.011 [c]	0.975±0.050 [b]	6.26±0.06 [c]
		15981	0.171±0.009 [d]	9.986±0.138 [c]	6.43±0.07 [d]
		132	0.057±0.003 [b]	0.958±0.037 [b]	5.93±0.02 [b]
		V329	0.172±0.000 [d]	10.788±0.199 [d]	6.50±0.03 [e]
		CH1368	0.014±0.001 [a]	0.027±0.002 [a]	2.33±0.01 [a]
			***	***	***
	S. epidermidis	F12	0.127±0.006	0.898±0.003	6.14±0.02
		CH48	0.080±0.006	0.034±0.002	2.02±0.06
			***	***	***
24 h	*S. aureus*	ISP479r	0.167±0.008 [d]	1.873±0.017 [c]	6.48±0.02 [c]
		15981	0.187±0.012 [e]	10.750±0.550 [d]	6.49±0.03 [c]
		132	0.064±0.003 [b]	0.806±0.001 [b]	6.04±0.05 [b]
		V329	0.132±0.000 [c]	11.042±0.361 [d]	6.48±0.04 [c]
		CH1368	-0.030±0.002 [a]	0.033±0.001 [a]	2.02±0.02 [a]
			***	***	***
	S. epidermidis	F12	0.142±0.011	0.901±0.056	6.34±0.01
		CH48	0.096±0.004	0.017±0.005	1.26±0.24
			***	***	***
