# Supplementary material for: Monitoring in Real Time the Formation and Removal of Biofilms from Clinical Related Pathogens Using an Impedance-Based Technology
Source: PLoS One. 2016 Oct 3;11(10):e0163966. doi: 10.1371/journal.pone.0163966 (PMC5047529; doi:10.1371/journal.pone.0163966)
Supplement: S2 Table — Differences were considered stable when two or more consecutive p-values were lower than 0.05. (PDF) [file pone.0163966.s004.pdf]

**Strain**	**Time** (h)	**Treatment**	**CI**(Mean ± SD)	**p-value**
S. aureus 15981	1	Control<br>LysH5	-0.055±0.0111<br>-0.050±0.0050	0.504
	2	Control<br>LysH5	-0.053±0.0092<br>-0.049±0.0040	0.540
	3	Control<br>LysH5	-0.044±0.0234<br>-0.031±0.0111	0.447
	4	Control<br>LysH5	-0.018±0.0017<br>-0.012±0.0027	**0.038**
	5	Control<br>LysH5	0.050±0.0165<br>0.038±0.0165	0.413
	6	Control<br>LysH5	0.085±0.0090<br>0.042±0.0010	**0.001**
	7	Control<br>LysH5	0.097±0.0003<br>0.045±0.0003	**0.000**
	8	Control<br>LysH5	0.110±0.0142<br>0.048±0.0005	**0.002**
	9	Control<br>LysH5	0.127±0.0094<br>0.049±0.0006	**0.000**
	10	Control<br>LysH5	0.129±0.0134<br>0.047±0.0016	**0.000**
S. epidermidis F12	1	Control<br>phi-IPLA7	-0.054±0.0151<br>-0.052±0.0067	0.893
	2	Control<br>phi-IPLA7	-0.053±0.0087<br>-0.053±0.0078	0.991
	3	Control<br>phi-IPLA7	-0.027±0.0066<br>-0.027±0.0065	0.819
	4	Control<br>phi-IPLA7	0.019±0.0049<br>0.006±0.0027	**0.012**
	5	Control<br>phi-IPLA7	0.025±0.0037<br>0.011±0.0015	**0.003**
	6	Control<br>phi-IPLA7	0.058±0.0027<br>0.043±0.0005	**0.001**
	7	Control<br>phi-IPLA7	0.064±0.0062<br>0.058±0.0006	0.219
	8	Control<br>phi-IPLA7	0.069±0.0022<br>0.048±0.0017	**0.000**
	9	Control<br>phi-IPLA7	0.076±0.0032<br>0.057±0.0029	**0.002**
	10	Control<br>phi-IPLA7<br>(MOI100)	0.081±0.0051<br>0.056±0.0026	**0.002**
